# Draft Whole-Genome Sequences of Periodontal Pathobionts *Porphyromonas gingivalis*, *Prevotella intermedia*, and *Tannerella forsythia* Contain Phase-Variable Restriction-Modification Systems

**DOI:** 10.1128/genomeA.01229-17

**Published:** 2017-11-16

**Authors:** Richard D. Haigh, Liam A. Crawford, Joseph D. Ralph, Joseph J. Wanford, Sonia R. Vartoukian, Karolin Hijazi, William Wade, Marco R. Oggioni

**Affiliations:** a Department of Genetics, University of Leicester, Leicester, United Kingdom; b Centre for Immunobiology, Blizard Institute, Barts and The London School of Medicine and Dentistry, Queen Mary University of London, United Kingdom; c School of Medicine & Dentistry, The University of Aberdeen Dental School and Hospital, Aberdeen, United Kingdom

## Abstract

Periodontal disease comprises mild to severe inflammatory host responses to oral bacteria that can cause destruction of the tooth-supporting tissue. We report genome sequences for 18 clinical isolates of *Porphyromonas gingivalis*, *Prevotella intermedia*, and *Tannerella forsythia*, Gram-negative obligate anaerobes that play a role in the periodontal disease process.

## GENOME ANNOUNCEMENT

Periodontal disease describes a range of mild to severe inflammatory oral bacterial infections that can ultimately cause destruction of the tooth-supporting tissues. Periodontitis affects 10 to 15% of the adult population worldwide (1). The host inflammation seen in periodontitis is provoked by oral bacteria and a number of species, including *Porphyromonas gingivalis*, *Prevotella intermedia*, and *Tannerella forsythia*, have been shown to be disease associated (2); *P. gingivalis*, in particular, is regarded as a keystone pathobiont subverting host defenses (3). Here, we describe the draft whole-genome sequences (WGS) of 18 anaerobic bacterial strains isolated from patients; the strains were selected from the culture collection of author W. Wade, obtained during previous studies. In those studies, subgingival plaque samples were collected from periodontal pockets >8 mm in depth in subjects with advanced periodontitis by means of a curette. Samples were cultured on fastidious anaerobe agar (FAA, Lab M) supplemented with 5% horse blood and incubated anaerobically for up to 7 days. *P. intermedia*, *T. forsythia*, and *P. gingivalis* strains were identified by 16S rRNA analysis. Genomic DNA isolated from all three species (Genomic DNA clean and concentrate kit, Zymo Research) was used to prepare libraries (Nextera DNA library preparation kit) which were analyzed on Illumina MiSeq. Sequence reads were quality controlled using Trimmomatic (4) and WGS assembled using SPAdes v3.6.2 (5). Genome size and assembly quality were assessed using QUAST v4.3 (6) (see Table 1).

**TABLE 1:** Sequence quality data for periodontal pathobiont draft whole-genome sequences

Strain	Species	Genome size (bp)	Contig no.	*N*_50_ (bp)	GC (%)	MLST	Comment	GenBank accession no.
WW414	*Prevotella intermedia*	2,620,156	62	129,786	43.4	NA^a^	pv-RMS^b^	NSMA00000000
WW855	*Prevotella intermedia*	2,688,123	137	51,409	43.5	NA	pv-RMS, *cfxA2*	NSLZ00000000
WW2834	*Prevotella intermedia*	2,804,910	126	70,737	43.3	NA	pv-RMS	NSLY00000000
WW2096	*Porphyromonas gingivalis*	2,333,958	116	48,508	48.4	Novel	pv-RMS	NSLX00000000
WW2842	*Porphyromonas gingivalis*	2,250,271	95	95,628	48.5	Novel	pv-RMS	NSLW00000000
WW2866	*Porphyromonas gingivalis*	2,314,500	122	43,452	48.5	Incomplete		NSLV00000000
WW2881	*Porphyromonas gingivalis*	2,478,925	484	23,327	48.2	Incomplete		NSLU00000000
WW2885	*Porphyromonas gingivalis*	2,402,406	196	36,927	48.5	Incomplete	pv-RMS	NSLT00000000
WW2903	*Porphyromonas gingivalis*	2,377,665	115	59,779	48.3	Novel		NSLS00000000
WW2931	*Porphyromonas gingivalis*	2,319,756	103	51,669	48.4	Incomplete		NSLR00000000
WW2952	*Porphyromonas gingivalis*	2,314,846	162	45,969	48.5	ST30	PgSL1 phage	NSLQ00000000
WW3039	*Porphyromonas gingivalis*	2,334,097	132	56,032	48.4	Novel		NSLN00000000
WW3040	*Porphyromonas gingivalis*	2,218,119	122	54,758	48.5	Novel		NSLP00000000
WW3102	*Porphyromonas gingivalis*	2,293,608	149	72,638	48.4	Incomplete	pv-RMS	NSLO00000000
WW5019	*Porphyromonas gingivalis*	2,307,097	149	76,761	48.4	ST30	pv-RMS	NSLM00000000
WW5127	*Porphyromonas gingivalis*	2,367,137	119	74,548	48.2	Novel		NSLL00000000
WW10960	*Tannerella forsythia*	3,312,685	98	101,673	47.2	NA		NSLK00000000
WW11663	*Tannerella forsythia*	3,300,179	140	79,152	47.1	NA		NSLJ00000000

a NA, not applicable.

b Phase-variable type I restriction-modification system.

Multilocus sequence typing (MLST) of the *P. gingivalis* WGS using pubMLST (pubmlst.org) identified two strains as sequence type 30 (ST30); however, six strains presented with novel STs, and the rest had incomplete MLST profiles (see Table 1). A core genome analysis of the *P. gingivalis* WGS, using the Harvest 1.0 program suite (http://harvest.readthedocs.io) (7), indicated that they all nest within the existing *P. gingivalis* genomes available in NCBI GenBank. WGS of all species were analyzed against the Comprehensive Antibiotic Resistance Database (https://card.mcmaster.ca/analyze) (8) to identify known and putative antimicrobial resistance genes. Two “perfect hits” were obtained, both in *P. intermedia* strain 885, against the *cfxA2* gene; this broad spectrum β-lactamase has been reported in several *Prevotella* spp. (9). Analysis of flanking sequence revealed the presence of a Tn*4555*-like sequence, from *Bacteroides fragilis*, suggesting horizontal acquisition (10). PHASTER (PHAge Search Tool Enhanced Release) (11) analysis of all WGS found just a single intact bacteriophage (33.8 kbp in length, with a G+C content of 48.78%, and encoding 36 proteins) in *P. gingivalis* WW2952.

Phase-variable type I restriction-modification systems (pv-RMS) were found in all of the *P. intermedia* genomes and in five of the *P. gingivalis* genomes (Table 1); similar pv-RMS were subsequently identified in *P. intermedia* and *P. gingivalis* genomes already in the GenBank database. A pv-RMS system found in *Streptococcus pneumoniae* has recently been shown to facilitate the epigenetic control of genes involved in virulence (12, 13). Structural similarities between the *S. pneumoniae* system and the pv-RMSs identified in *P. intermedia* and *P. gingivalis* raise the possibility that epigenetic regulatory mechanisms may also play a role in periodontal disease.

### Accession number(s).

These whole-genome shotgun sequences have been deposited in GenBank and the versions described in this paper are the first versions (see Table 1 for full details).
